# AIDS and non-AIDS severe morbidity associated with hospitalizations among HIV-infected patients in two regions with universal access to care and antiretroviral therapy, France and Brazil, 2000–2008: hospital-based cohort studies

**DOI:** 10.1186/1471-2334-14-278

**Published:** 2014-05-21

**Authors:** Paula Mendes Luz, Mathias Bruyand, Sayonara Ribeiro, Fabrice Bonnet, Ronaldo Ismério Moreira, Mojgan Hessamfar, Dayse Perreira Campos, Carine Greib, Charles Cazanave, Valdilea Gonçalves Veloso, François Dabis, Beatriz Grinsztejn, Geneviève Chêne

**Affiliations:** 1 Instituto de Pesquisa Clínica Evandro Chagas, Fundação Oswaldo Cruz, Rio de Janeiro 21040, Rio de Janeiro, Brasil; 2 Centre Hospitalier Universitaire de Bordeaux, Pôle de Santé Publique, COREVIH Aquitaine, Bordeaux F-33000, France; 3 INSERM, ISPED, Centre Inserm U897- Epidemiologie-Biostatistique & CIC-EC7, Bordeaux F-33000, France; 4 Centre Hospitalier Universitaire de Bordeaux, Hôpital Haut-Lévèque, Pessac F-33600, France; 5 Centre Hospitalier Universitaire de Bordeaux, Hôpital Pellegrin, Bordeaux F-33000, France

**Keywords:** HIV, AIDS, Antiretroviral therapy, Severe morbidity, Hospitalization, Cohort study, Brazil, France

## Abstract

**Background:**

In high-income settings, the spectrum of morbidity and mortality experienced by Human Immunodeficiency Virus (HIV)-infected individuals receiving combination antiretroviral therapy (cART) has switched from predominantly AIDS-related to non-AIDS-related conditions. In the context of universal access to care, we evaluated whether that shift would apply in Brazil, a middle-income country with universal access to treatment, as compared to France.

**Methods:**

Two hospital-based cohorts of HIV-infected individuals were used for this analysis: the ANRS CO3 Aquitaine Cohort in South Western France and the Evandro Chagas Research Institute (IPEC) Cohort of the Oswaldo Cruz Foundation in Rio de Janeiro, Brazil. Severe morbid events (AIDS- and non-AIDS-related) were defined as all clinical diagnoses associated with a hospitalization of ≥48 hours. Trends in the incidence rate of events and their determinants were estimated while adjusting for within-subject correlation using generalized estimating equations models with an auto-regressive correlation structure and robust standard errors.

**Result:**

Between January 2000 and December 2008, 7812 adult patients were followed for a total of 41,668 person-years (PY) of follow-up. Throughout the study period, 90% of the patients were treated with cART. The annual incidence rate of AIDS and non-AIDS events, and of deaths significantly decreased over the years, from 6.2, 21.1, and 1.9 AIDS, non-AIDS events, and deaths per 100 PY in 2000 to 4.3, 14.9, and 1.5/100 PY in 2008. The annual incidence rates of non-AIDS events surpassed that of AIDS-events during the entire study period. High CD4 cell counts were associated with a lower incidence rate of AIDS and non-AIDS events as well as with lower rates of specific non-AIDS events, such as bacterial, hepatic, viral, neurological, and cardiovascular conditions. Adjusted analysis showed that severe morbidity was associated with lower CD4 counts and higher plasma HIV RNAs but not with setting (IPEC versus Aquitaine).

**Conclusions:**

As information on severe morbidities for HIV-infected patients remain scarce, data on hospitalizations are valuable to identify priorities for case management and to improve the quality of life of patients with a chronic disease requiring life-long treatment. Immune restoration is highly effective in reducing AIDS and non-AIDS severe morbid events irrespective of the setting.

## Background

Currently, individuals infected by the human immunodeficiency virus (HIV) and treated with combination antiretroviral therapy (cART) live longer than ever in high-income countries [1]. The spectrum of the disease has switched in this environment from predominantly AIDS-related mortality and morbidity to non-AIDS-related conditions, especially severe infections, end-stage liver disease, cardiovascular disease, and malignancies [2-5]. In Brazil, a middle-income country with widely available cART, AIDS-related conditions remain an important cause of mortality and morbidity, while an increased representation of non-AIDS defining conditions has recently been reported [6,7].

Whereas causes of death are now well described [2,4], severe morbidity has been more difficult to accurately study and data remain scarce about their distribution and determinants over time. Observational hospital-based cohorts from Europe and North America have reported causes of hospitalization in the cART era as a proxy for severe morbidity, especially in the context of universal access to care. Indeed, updated information on severe disease burden can inform HIV clinicians as to the necessary efforts for screening and prevention and can be used for monitoring patients with organ-specific specialists. Policy-makers can also use such information to project future areas of care needs and associated costs. Observational hospital-based cohort studies have shown that HIV-infected individuals continue to be hospitalized at high rates [3,8,9], with a shift towards non-AIDS severe morbid events [10]. Despite the fast evolution of cART coverage including in resource-constrained settings, there have been only few reports from these areas over the past ten years. Severe morbidity in resource-limited settings might also be characterized by the same shift towards non-AIDS events, though patients are exposed to specific tropical infectious diseases [11].

Our aim was to describe and contrast severe morbidity trends and determinants among HIV-infected individuals followed in two geographically contrasting settings (France and Brazil) with universal access to treatment and care. To this end, we studied severe morbidity over a 9-year period in two hospital-based cohorts of HIV-infected individuals: the ANRS C03 Aquitaine (Aquitaine) in South Western France and the Evandro Chagas Research Institute (IPEC) Cohort of the Oswaldo Cruz Foundation in Rio de Janeiro, Brazil.

## Methods

### Study population

The ANRS CO3 Aquitaine Cohort is an open prospective cohort of HIV-1 infected patients followed in nine public hospitals of the Aquitaine region in Southwestern France. The cohort was initiated in 1987 and includes adults with documented HIV infection regardless of clinical stage and informed consent. Details of the cohort can be found elsewhere [12]. The ANRS CO3 Aquitaine Cohort was approved by the French Commission Nationale de L'Informatique et des Libertés (88–84, 88–142, DR-2010-109). Participants provided written informed consent and the study is conducted in compliance with the Helsinki Declaration.

The Evandro Chagas Clinical Research Institute (IPEC) has provided care to HIV-infected patients in Rio de Janeiro since 1986. An observational, longitudinal, clinical database has been maintained on patients receiving primary HIV care in the clinic since 1998. Longitudinal data are updated regularly using outpatient and inpatient clinical documentation, laboratory testing results, and pharmacy records. Prescription of cART (drug, dates of use, and dose) is documented by the medical provider and support staff in the clinical records. Trained abstractors record all this information onto standardized forms for processing. Details of the cohort have been described elsewhere [13]. The IPEC cohort was approved by the Evandro Chagas Clinical Research Ethics Committee (043/2010, 0032.0.009.000-10). Participants provided written informed consent and the study is conducted in compliance with the Helsinki Declaration.

The sample selected for this analysis included all adult patients (age ≥ 18 years) followed in each cohort from 2000 to 2008.

### Definitions

A severe morbid event was defined as a clinical diagnosis associated with a hospitalization with at least 48 hours of duration. For each hospitalization, all diagnosis listed in the hospital records at hospitalization discharge were counted as separate severe morbid events. That is, one hospitalization could be associated with several clinical diagnoses, and, in this case, each different diagnosis was taken into account. Dates of admission and discharge were captured among both discharged patients and those who died during hospital stay.

The ANRS C03 Aquitaine Cohort used a simplified version of the International Classification of Diseases (ICD-10) and the IPEC Cohort used ICD-10 to code the clinical diagnoses. As some codes could correspond to several disease categories, we considered a hierarchical classification with a decreasing order of priority considering AIDS-events, non-AIDS malignancies, infections and systemic events. Signs and syndromes were considered as events if they were not reported along with other diagnoses during a specific hospitalization. Hospitalizations due to a non-morbid cause (check-ups, chemotherapy, drug withdrawal programme, and more generally Z codes of the ICD-10 classification), whatever the duration of the stay, were not considered in our definition of severe morbidity and were not included in this analysis. All events retrieved from the database were reviewed and validated by two clinicians highly experienced in the management of HIV infection, one clinician from Brazil and another from France. The lists of codes allocated to the different disease categories by each cohort were then compared and all discrepancies were discussed until a consensus was reached.

Severe morbid events were classified under 22 disease categories as follows: (1) AIDS, (2) Non-AIDS malignancies, (3) In situ malignancies, (4) Cardiovascular, (5) Hepatic, (6) Digestive, (7) Psychiatric, (8) Hematological, (9) Renal, (10) Endocrine, (11) Bacterial, (12) Viral, (13) Parasitic, (14) Dermatological, (15) Gynecological, (16) Neurological, (17) Ophthalmological, (18) Respiratory, (19) Rheumatologic, (20) Trauma, (21) Signs and syndromes, and (22) Other.

Age from birth was updated yearly for each participant. Presumed mode of HIV exposure was classified as men who have sex with men (MSM), injection drug use (IDU), heterosexual transmission, other, and unknown. Yearly CD4 cell counts and plasma HIV RNAs were defined as the mean value of all measurements taken in a specific calendar year. CD4 cell counts were categorized into >500, 500–351, 350–201, 200–51, ≤ 50 cells/mm^3^. For consistency across years, 400 copies/ml of plasma HIV RNA was defined as the limit of detection. Plasma HIV RNA was categorized into ≤ 400, 401–3000, 3001–10000, 10001–100000, >100000 copies/ml. Time since first HIV positive test was calculated for each calendar year. cART was defined as two nucleoside reverse transcriptase inhibitors plus either one non-nucleoside reverse transcriptase inhibitor or one protease-inhibitor (boosted or not). Hepatitis B infection was defined by the presence of hepatitis B surface antigen (HBsAg) while hepatitis C infection was defined by the presence of hepatitis C antibodies.

### Analyses

Descriptive analyses of the demographic and clinical characteristics of the study patients were conducted, including age, gender, mode of HIV transmission, CD4 cell count, plasma HIV RNA, time since first HIV positive test, ever use of cART, and hepatitis B and C co-infections.

Each patient contributed to multiple years of observation, one for each calendar year. Patients could enroll in the cohort at any time preceding or during the study period (1 January 2000 to 31 December 2008), and thus the number of person-years was not constant across patients or years. Within each year, we calculated the number of days of follow-up. If a patient enrolled in a given year, the number of days prior to enrolment was excluded from the count of number of days of follow up for that year. Individuals were not censored after an episode of hospitalization, that is, readmissions were also studied, irrespective of the reason for hospitalization. The end of follow-up was defined as the last contact with the clinic, 31 December 2008, or death, whichever occurred first.

We estimated the rate of severe morbid events overall and by category for each calendar year using as numerator the number of severe morbid events and as denominator the person-years (PY) of follow-up. To assess trends over time, calendar year (2000 to 2008) was included in the regression model and the significance of the per year increase coefficient was tested. Age, CD4 cell count, plasma HIV RNA, time since first HIV positive test, ever use of cART, and hepatitis B and C co-infection were updated for each year of follow-up. Adjusted incidence rate ratios were used to measure the degree of association of demographic and clinical factors with the overall incidence rate of AIDS, death, and non-AIDS events, as well as the specific incidence rate of non-AIDS events categories.

We used generalized estimating equations (GEE) models with an auto-regressive correlation matrix and robust standard errors to adjust for the clustered nature of the data here represented by the multiple observation years for each patient (assumption of independence between clusters). The specified working correlation structure (i.e. the auto-regressive correlation matrix) describes how the responses (annual rate of events) within clusters (patients) are related to each other. GEE models have been extensively used for data in which the responses are correlated [14,15]. The R Project for Statistical Computing (version 2.15.2) was used for all analyses.

## Results

Between January 2000 and December 2008, 7812 patients were followed for a total of 41,668 person-years (PY) of follow-up, 29,866 PY for Aquitaine and 11,802 PY for IPEC. The mean follow-up time per patient for the two cohorts combined was 5.3 years (median 5.4 years, interquartile range [IQR] 2.3-9.0 years). Approximately two-thirds of the study population was from Aquitaine and its mean follow-up time per patient was 6.0 years (median 6.9 years, IQR 3.2-9.0 years), compared to 4.2 years (median 3.3 years, IQR 1.4-7.4 years) for IPEC.

Demographic and clinical characteristics of the study population varied over time (Table 1). The proportion of patients in the age groups 50–59 and ≥ 60 years of age increased from 9.2% and 4.2% in 2000 to 17.8% and 8.2% in 2008, respectively. The relative frequency of heterosexual contact as the mode of HIV transmission increased while injection drug use decreased over time. Patients’ immunological profile at a given year significantly improved with time and half of the study population reaching a CD4 cell count >500 cells/mm^3^ in 2008; in addition two-thirds of the population had undetectable plasma HIV RNA in 2008. The prevalence of hepatitis C decreased over time. Ageing, decreased proportion of IDUs, and improved immunological profile were observed in the two cohorts when evaluated separately, though trends were more pronounced in the Aquitaine Cohort (see Additional file 1: Tables S1 and S2). In IPEC, an increased proportion of males was observed over time. Throughout the study period, the proportion of patients diagnosed with HIV infection for less than one year remained stable (10%), as was the use of cART (90%), and the co-infection with hepatitis B virus (6%).

**Table 1:** **Demographic and clinical characteristics** (**number** [**percent**]) **of the study population by year**, **ANRS CO3 Aquitaine and IPEC**/**FIOCRUZ cohorts combined**

	**2000**	**2001**	**2002**	**2003**	**2004**	**2005**	**2006**	**2007**	**2008**
Number of patients	3911	4165	4375	4568	4898	5267	5621	5916	6144
Age in years^a^									
< 30	430 (11.0)	419 (10.1)	364 (8.3)	336 (7.4)	325 (6.6)	384 (7.3)	426 (7.6)	456 (7.7)	483 (7.9)
30-39	1830 (46.8)	1808 (43.4)	1787 (40.8)	1690 (37)	1614 (33)	1563 (29.7)	1501 (26.7)	1494 (25.3)	1462 (23.8)
40-49	1127 (28.8)	1316 (31.6)	1505 (34.4)	1715 (37.5)	1987 (40.6)	2183 (41.4)	2422 (43.1)	2547 (43.1)	2603 (42.4)
50-59	359 (9.2)	435 (10.4)	518 (11.8)	597 (13.1)	700 (14.3)	811 (15.4)	905 (16.1)	982 (16.6)	1093 (17.8)
> = 60	165 (4.2)	187 (4.5)	201 (4.6)	230 (5)	272 (5.6)	326 (6.2)	367 (6.5)	437 (7.4)	503 (8.2)
Gender									
Male	2725 (69.7)	2879 (69.1)	3039 (69.5)	3175 (69.5)	3418 (69.8)	3679 (69.9)	3898 (69.3)	4096 (69.2)	4256 (69.3)
Mode of HIV transmission^b^									
Heterosexual	1129 (28.9)	1274 (30.6)	1371 (31.3)	1464 (32)	1613 (32.9)	1798 (34.1)	1994 (35.5)	2176 (36.8)	2328 (37.9)
MSM	1383 (35.4)	1471 (35.3)	1575 (36)	1682 (36.8)	1817 (37.1)	1963 (37.3)	2102 (37.4)	2221 (37.5)	2320 (37.8)
IDU	1011 (25.9)	996 (23.9)	984 (22.5)	955 (20.9)	965 (19.7)	947 (18)	933 (16.6)	909 (15.4)	858 (14)
Other	145 (3.7)	150 (3.6)	151 (3.5)	155 (3.4)	155 (3.2)	157 (3)	165 (2.9)	161 (2.7)	166 (2.7)
Unknown	243 (6.2)	274 (6.6)	294 (6.7)	312 (6.8)	348 (7.1)	402 (7.6)	427 (7.6)	449 (7.6)	472 (7.7)
CD4 cell count^c^									
> 500	1306 (38.3)	1412 (39.5)	1580 (41)	1646 (41.9)	1768 (40.7)	1996 (41.9)	2274 (44.9)	2457 (45.3)	2720 (47.2)
351-500	787 (23.1)	866 (24.2)	920 (23.9)	924 (23.5)	1075 (24.7)	1167 (24.5)	1225 (24.2)	1375 (25.4)	1453 (25.2)
201-350	777 (22.8)	783 (21.9)	795 (20.6)	852 (21.7)	931 (21.4)	963 (20.2)	997 (19.7)	985 (18.2)	1017 (17.7)
51-200	427 (12.5)	410 (11.5)	449 (11.7)	414 (10.5)	446 (10.3)	502 (10.5)	459 (9.1)	501 (9.2)	479 (8.3)
<= 50	114 (3.3)	107 (3)	107 (2.8)	94 (2.4)	128 (2.9)	132 (2.8)	115 (2.3)	103 (1.9)	93 (1.6)
Plasma HIV RNA^c^									
<= 400	1151 (33.8)	1324 (37.3)	1628 (42.3)	1859 (47.3)	2100 (48.8)	2514 (53.5)	2908 (58.5)	3348 (63.1)	3898 (68.4)
401-3000	749 (22)	795 (22.4)	689 (17.9)	598 (15.2)	613 (14.3)	604 (12.8)	557 (11.2)	511 (9.6)	518 (9.1)
3001-10000	475 (13.9)	451 (12.7)	445 (11.6)	409 (10.4)	393 (9.1)	396 (8.4)	345 (6.9)	354 (6.7)	318 (5.6)
10001-100000	725 (21.3)	716 (20.2)	781 (20.3)	789 (20.1)	871 (20.3)	862 (18.3)	787 (15.8)	805 (15.2)	721 (12.6)
> 100000	308 (9)	267 (7.5)	306 (8)	277 (7)	323 (7.5)	325 (6.9)	378 (7.6)	288 (5.4)	246 (4.3)
≤ 1 year since first HIV + test^d^									
Yes	439 (11.2)	350 (8.4)	337 (7.7)	316 (6.9)	316 (6.5)	421 (8.0)	493 (8.8)	496 (8.4)	501 (8.2)
On cART^e^									
Yes	3506 (89.6)	3724 (89.4)	3932 (89.9)	4101 (89.8)	4393 (89.7)	4684 (88.9)	4913 (87.4)	5197 (87.8)	5508 (89.6)
Hepatitis B^f^									
Yes	215 (6.1)	235 (6.2)	249 (6.2)	258 (6.1)	280 (6.2)	305 (6.3)	331 (6.4)	348 (6.4)	367 (6.6)
Hepatitis C^g^									
Yes	949 (26.7)	968 (25.5)	1013 (25.2)	1022 (24.2)	1027 (22.6)	1008 (20.5)	1018 (19.4)	999 (18.2)	978 (17.3)

^a^Age from birth was updated yearly for each participant.

^b^Presumed mode of HIV exposure was classified as men who have sex with men (MSM), injection drug use (IDU), heterosexual transmission, other, and unknown.

^c^Yearly CD4 cell counts and plasma HIV RNAs were defined as the mean value of all measurements taken in a specific calendar year.

^d^Time since first HIV + test was calculated for each calendar year.

^e^Combination antiretroviral therapy (cART) was defined as two nucleoside reverse transcriptase inhibitors plus either one non-nucleoside reverse transcriptase inhibitor or one protease-inhibitor (boosted or not).

^f^Hepatitis B infection was defined by the presence of hepatitis B surface antigen (HBsAg).

^g^Hepatitis C infection was defined by the presence of hepatitis C antibodies.

During the entire study period, hospitalization for a severe morbid event occurred in a pool of 2238 (28.6%) participants, 1473 (29.5%) from Aquitaine and 765 (27.1%) from IPEC. A total of 4689 hospitalizations were recorded, 3049 (65.0%) in Aquitaine and 1640 (35.0%) in IPEC. One severe morbid event was reported in 2348 hospitalizations (50.1%), two morbid events in 1359 hospitalizations (29.0%), and three in 599 hospitalizations (12.8%). In 383 (8.2%) hospitalizations more than three severe morbid events were reported. Among those who were hospitalized (2238/7812, 28.6%), 8696 severe morbid events were reported, 5963 (68.6%) in Aquitaine and 2733 (31.4%) in IPEC. There were 310 deaths reported, a 6.6% fatality ending hospitalizations.

Over the entire study period, the incidence rate of severe morbid events was 21.5/100 PY (95% confidence interval (95% CI): 20.3 to 22.8/100 PY) overall, 20.5/100 PY (95% CI 19.1 to 22.0/100 PY) in Aquitaine and 24.0/100 PY (95% CI 21.8 to 26.4/100 PY) in IPEC. The annual incidence rate of severe morbid events significantly decreased from 27.3/100 PY in 2000 to 19.2/100 PY in 2008 (p < 0.001, Table 2). This downward trend was observed for both AIDS and non-AIDS events. The annual incidence rate of non-AIDS events surpassed that of AIDS-events during the entire study period. These trends were observed in both cohorts though the ratio of annual incidence rates of non-AIDS to AIDS events was much higher in the Aquitaine Cohort (up to 6:1) than in the IPEC Cohort (always <2:1).

**Table 2:** **Annual incidence rates per 100 person**-**years of severe morbid events**, **AIDS and non**-**AIDS events**, **and deaths**, **overall and by cohort** (**ANRS CO3 Aquitaine and IPEC**/**FIOCRUZ cohorts**)

	**2000**	**2001**	**2002**	**2003**	**2004**	**2005**	**2006**	**2007**	**2008**	**P-value**
**Overall**										
**Severe morbid event**	27.3	26.4	20.5	21.2	20.3	19.4	18.0	19.1	19.2	< 0.01
**AIDS**	6.2	5.7	5.0	4.4	5.1	4.6	3.6	3.8	4.3	< 0.01
**Non-AIDS**	21.1	20.7	15.5	16.7	15.2	14.8	14.4	15.3	14.9	< 0.01
**Death**	1.9	2.7	2.3	2.2	2.5	2.6	2.0	2.0	1.5	< 0.01
**ANRS CO3 Aquitaine**										
**Severe morbid event**	26.2	25.2	18.7	18.9	18.7	17.6	17.8	20.0	18.2	< 0.01
**AIDS**	4.7	4.4	3.1	2.7	2.9	2.5	1.9	2.5	2.0	< 0.01
**Non-AIDS**	21.6	20.8	15.6	16.2	15.8	15.2	15.9	17.5	16.2	0.04
**Death**	1.6	2.3	2.2	1.7	1.6	1.9	1.5	1.3	1.1	< 0.01
**IPEC/FIOCRUZ**										
**Severe morbid event**	31.4	30.5	26.2	27.8	24.6	23.9	18.3	17.3	20.9	< 0.01
**AIDS**	12.0	10.2	11.1	9.5	10.9	10.1	7.3	6.4	8.2	< 0.01
**Non-AIDS**	19.4	20.4	15.1	18.3	13.6	13.7	11.0	10.8	12.7	< 0.01
**Death**	3.0	4.0	2.6	3.6	4.7	4.2	3.0	3.3	2.3	0.09

Annual mortality rates also decreased over time. Eight hundred and ninety one patients died during the study period yielding a mortality rate that decreased from 1.9/100 PY in 2000 to 1.5/100 PY in 2008 (p < 0.01). In Aquitaine, 498 patients died during the study period and the mortality rate decreased from 1.6/100 PY in 2000 to 1.1/100PY in 2008 (p < 0.01). In IPEC, 393 deaths occurred yielding a higher mortality rate that also decreased from 3.0/100 PY in 2000 to 2.3/100 PY in 2008 although the difference did not reach statistical significance (p = 0.09).

The annual incidence rates of the 21 non-AIDS disease categories are displayed in Table 3. Bacterial infection was the most frequent cause of non-AIDS events with a total of 1602 events yielding a stable incidence rate over time ranging between 3.5 and 4.5 events per 100 PY (p = 0.12). Although similar for each cohort when evaluated separately (see Additional file 1: Table S3 and Table S4), the incidence was much higher in IPEC with a maximum of 9.0 events per 100 PY in 2001 while in Aquitaine the maximum was 3.9 events per 100 PY in 2000. Following bacterial infection and by decreasing order of importance, the following disease categories were: psychiatric diseases, hepatic diseases, viral, hematological, neurological, digestive, cardiovascular, and parasitic diseases. In Aquitaine, psychiatric diseases held the second position while cardiovascular events were the second most frequent event category in IPEC (see Additional file 1: Tables S3 and S4). Psychiatric, viral, neurological, digestive, parasitic and dermatological diseases showed a statistically significant decreasing trend over the study period in the two cohorts combined (Table 3). The incidence rate of non-AIDS defining malignancies ranged between 0.1 and 0.5 events per 100 PY but did not decrease over time (p = 0.73).

**Table 3:** **Annual incidence rates per 100 person-years of non-AIDS events ranked from most to least frequent** (**ANRS CO3 Aquitaine and IPEC**/**FIOCRUZ cohorts combined**)

		**2000**	**2001**	**2002**	**2003**	**2004**	**2005**	**2006**	**2007**	**2008**	**P-value**	**Total # of events**
**1**	**Bacterial**	4.50	4.35	3.69	3.93	3.91	3.52	3.65	3.73	3.64	0.12	1602
**2**	**Psychiatric**	2.39	1.89	1.64	1.70	2.09	1.69	1.85	1.42	1.34	< 0.01	727
**3**	**Hepatic**	1.21	1.97	1.12	1.14	1.29	1.56	1.37	1.35	1.36	0.61	572
**4**	**Viral**	2.33	1.32	1.51	1.16	1.14	0.95	0.69	0.71	1.06	< 0.01	481
**5**	**Hematological**	0.90	1.63	1.27	1.23	1.34	1.01	1.00	0.93	1.15	0.51	479
**6**	**Neurological**	1.40	1.89	1.10	1.30	0.94	1.07	0.79	0.60	0.99	< 0.01	450
**7**	**Digestive**	1.70	1.48	0.88	1.21	0.86	0.99	1.00	0.76	0.79	< 0.01	433
**8**	**Cardiovascular**	0.93	1.19	0.76	0.91	0.70	0.64	0.88	0.98	1.10	0.82	375
**9**	**Parasitic**	1.15	0.83	0.66	1.07	0.79	0.49	0.52	0.66	0.49	< 0.01	298
**10**	**Signs/syndromes**	0.90	0.67	0.24	0.40	0.40	0.60	0.42	0.73	0.35	0.32	215
**11**	**Renal**	0.63	0.26	0.51	0.35	0.37	0.45	0.44	0.76	0.49	0.26	201
**12**	**Rheumatologic**	0.41	0.39	0.37	0.21	0.22	0.39	0.46	0.62	0.39	0.24	163
**13**	**Endocrine**	0.44	0.57	0.22	0.42	0.44	0.47	0.23	0.42	0.30	0.33	160
**14**	**Respiratory**	0.63	0.44	0.56	0.37	0.15	0.23	0.25	0.49	0.41	0.22	160
**15**	**Dermatological**	0.58	0.52	0.39	0.47	0.26	0.29	0.27	0.35	0.25	< 0.01	150
**16**	**Malignancies**	0.36	0.47	0.17	0.51	0.09	0.16	0.29	0.38	0.46	0.73	134
**17**	**Other**	0.44	0.34	0.27	0.23	0.13	0.14	0.06	0.20	0.11	< 0.01	83
**18**	**Ophthalmological**	0.08	0.39	0.02	0.02	0.09	0.02	0.08	0.09	0.11	0.22	40
**19**	**Trauma**	0.08	0.00	0.10	0.02	0.00	0.04	0.13	0.05	0.11	0.25	26
**20**	**Gynecological**	0.03	0.08	0.02	0.02	0.02	0.06	0.00	0.02	0.04	0.52	13
**21**	**In situ neoplasia**	0.03	0.00	0.00	0.02	0.00	0.00	0.00	0.00	0.00	NA	2

The three most frequent diagnoses within the AIDS category and specific non-AIDS categories are shown in Table 4 for both cohorts combined and in the Additional file 1: Table S5 and Table S6 for Aquitaine and IPEC Cohorts, respectively. Overall and in each cohort, the two most frequent diagnoses within the AIDS category were pneumocystosis and toxoplasmosis, while tuberculosis held the third position overall and in IPEC Cohort but not in Aquitaine Cohort. Bacterial pneumonia and sepsis were the most frequent diagnoses within bacterial infections while among psychiatric diseases the most frequent diagnoses were depression, drug abuse and anorexia. Overall, the top three diagnoses within cardiovascular diseases were hypertension, thrombosis, and cerebral infarction. Lung cancer and Hodgkin lymphoma were among the most frequent diagnoses within the non-AIDS malignancy category.

**Table 4:** **Three most frequent diagnoses within the AIDS and specific non**-**AIDS severe morbid events categories** (**ANRS CO3 Aquitaine and IPEC**/**FIOCRUZ cohorts combined**, **2000**–**2008**)

**Diagnosis (ICD-10 code)**	**N/Total N (%)**
**AIDS**	
Pneumocystosis (B59)	278/1932 (14.4)
Toxoplasmosis (B58.2)	161/1932 (8.3)
Tuberculosis (A15.3)	146/1932 (7.6)
**Non-AIDS**	
**Bacterial**	
Bacterial pneumonia (J15.9)	289/1602 (18.0)
Sepsis (A41.9)	204/1602 (12.7)
Pneumonia, other specified infectious organisms (J16.8)	127/1602 (7.9)
**Psychiatric**	
Depressive episode (F32.9)	190/727 (26.1)
Drug abuse (F19.9)	142/727 (19.5)
Anorexia (R63.0)	73/727 (10.0)
**Hepatic**	
Hepatic failure (K72.9)	100/572 (17.5)
Toxic liver disease with acute hepatitis (K71.2)	85/572 (14.9)
Ascites (R18)	61/572 (10.7)
**Viral**	
Acute bronchitis (J20.9)	71/481 (14.8)
Zoster infection (B02.9)	52/481 (10.8)
Gastroenteritis (A09)	46/481 (9.6)
**Neurological**	
Headache (R51)	76/450 (16.9)
Dizziness (R42)	44/450 (9.8)
Epilepsy (G40.9)	43/450 (9.6)
**Cardiovascular**	
Hypertension (I10)	39/375 (10.4)
Thrombosis (I82.9)	32/375 (8.5)
Cerebral infarction (I63.9)	29/375 (7.7)
**Parasitic**	
Candidal stomatitis (B37.0)	181/298 (60.7)
Mycosis (B49)	23/298 (7.7)
Candidiasis of other sites (B37.8)	9/298 (3.0)
**Non-AIDS malignancy**	
Lung cancer (C34.9)	40/134 (29.9)
Unspecified cancer (C80)	23/134 (17.2)
Hodgkin lymphoma (C81.9)	16/134 (11.9)

Adjusted incidence rate ratios quantifying the degree of association of demographic and clinical factors with the incidence rate of AIDS and non-AIDS events are shown in Table 5. Immunodeficiency was found to be associated with the severe morbid events. As expected, the association with low CD4 cell count was strong for AIDS events with a dose–response pattern. Albeit the magnitude of the association was smaller, lower CD4 cell counts were also associated with a higher incidence rate of non-AIDS events, of specific non-AIDS events, and of deaths (Table 5). Higher plasma HIV RNA, less than 1 year since first HIV positive test and use of cART were significantly associated with a higher incidence rate of AIDS events.

**Table 5:** **Adjusted incidence rate ratios and 95**% **confidence intervals for factors associated with AIDS**-**related events**, **non**-**AIDS**-**related events**, **and deaths** (**ANRS CO3 Aquitaine and IPEC**/**FIOCRUZ cohorts combined**, **2000**–**2008**)

	**AIDS-related**	**Non-AIDS**			
		**All**	**Bacterial**	**Psychiatric**	**Hepatic**
Cohort: IPEC (vs. Aquitaine)	1.09 (0.91, 1.3)	**0.53 (0.46, 0.62)**	1.17 (0.94, 1.47)	**0.37 (0.24, 0.55)**	0.78 (0.52, 1.17)
Calendar year (per year)	1.03 (0.99, 1.06)	1.02 (1.0, 1.05)	1.03 (1.0, 1.07)	1.04 (0.98, 1.09)	1.05 (0.98, 1.12)
Age (in years)					
<30 years	1 (Ref.)	1 (Ref.)	1 (Ref.)	1 (Ref.)	1 (Ref.)
30-39	0.95 (0.72, 1.26)	1.05 (0.83, 1.33)	1.34 (0.98, 1.83)	1.28 (0.76, 2.17)	0.8 (0.39, 1.65)
40-49	0.81 (0.61, 1.08)	1.12 (0.88, 1.44)	1.18 (0.85, 1.64)	1.18 (0.7, 2.01)	1.52 (0.78, 2.99)
50-59	0.59 (0.42, 0.83)	1.24 (0.94, 1.65)	1.13 (0.78, 1.64)	0.95 (0.5, 1.78)	1.48 (0.67, 3.28)
≥60	0.93 (0.61, 1.4)	**2.16 (1.58, 2.97)**	**1.9 (1.24, 2.89)**	1.14 (0.54, 2.38)	**3.09 (1.13, 8.49)**
Sex: Female (vs. Male)	0.9 (0.76, 1.07)	1.01 (0.88, 1.15)	0.95 (0.79, 1.15)	1.06 (0.81, 1.37)	0.98 (0.63, 1.52)
IDU: Yes (vs. No)	1.03 (0.78, 1.36)	**1.42 (1.18, 1.7)**	1.01 (0.75, 1.34)	**3.14 (2.29, 4.3)**	1.49 (0.89, 2.49)
CD4 cell count (cells/mm^3^)					
>500	1 (Ref.)	1 (Ref.)	1 (Ref.)	1 (Ref.)	1 (Ref.)
351-500	**3.47 (2.19, 5.51)**	**1.28 (1.07, 1.53)**	1.24 (0.95, 1.61)	0.96 (0.67, 1.38)	**2.42 (1.44, 4.08)**
201-350	**9.05 (5.91, 13.88)**	**2.25 (1.89, 2.67)**	**1.85 (1.43, 2.4)**	**1.98 (1.44, 2.73)**	**4.87 (2.97, 7.99)**
51-200	**31.83 (20.81, 48.71)**	**4.34 (3.59, 5.24)**	**3.94 (2.95, 5.26)**	**2.59 (1.76, 3.79)**	**9.52 (5.56, 16.29)**
≤50	**111.45 (70.13, 177.11)**	**10.99 (8.66, 13.94)**	**9.14 (6.37, 13.12)**	**4.98 (2.96, 8.37)**	**22.31 (11.27, 44.15)**
HIV viral load (copies/mL)					
≤400	1 (Ref.)	1 (Ref.)	1 (Ref.)	1 (Ref.)	1 (Ref.)
401-3000	**1.58 (1.14, 2.17)**	**1.3 (1.09, 1.54)**	**1.41 (1.09, 1.83)**	1.08 (0.75, 1.57)	1.28 (0.76, 2.17)
3001-10000	**1.6 (1.11, 2.29)**	**1.45 (1.18, 1.76)**	**1.12 (0.83, 1.52)**	**1.87 (1.25, 2.81)**	1.71 (0.86, 3.4)
10001-100000	**2.3 (1.76, 3.01)**	**1.76 (1.5, 2.06)**	**1.96 (1.54, 2.49)**	**2.02 (1.44, 2.84)**	1.53 (0.96, 2.43)
>100000	**4.39 (3.33, 5.8)**	**2.61 (2.15, 3.16)**	**2.79 (2.12, 3.68)**	**2.89 (1.93, 4.33)**	**2.18 (1.21, 3.92)**
≤1 year since HIV + test: Yes (vs. No)	**3.87 (3.19, 4.71)**	**1.99 (1.69, 2.35)**	**1.51 (1.16, 1.97)**	1.04 (0.65, 1.67)	**2.74 (1.84, 4.1)**
On cART: Yes (vs. No)	**2.15 (1.23, 3.74)**	**2.5 (1.82, 3.44)**	**1.58 (1.04, 2.41)**	**2.21 (1.12, 4.38)**	**3.72 (1.5, 9.2)**
Hepatitis B positive (vs. not)	1.03 (0.77, 1.39)	1.17 (0.93, 1.47)	1.23 (0.9, 1.68)	0.92 (0.57, 1.5)	**1.93 (1.16, 3.18)**
Hepatitis C positive (vs. not)	0.95 (0.74, 1.23)	**1.7 (1.45, 2.0)**	**1.91 (1.52, 2.4)**	**1.73 (1.29, 2.32)**	**5.18 (3.29, 8.14)**
	**Non-AIDS**				**Death**
	**Viral**	**Neurological**	**Cardiovascular**	**Parasitic**	
Cohort: IPEC (vs. Aquitaine)	**0.52 (0.38, 0.7)**	**0.06 (0.03, 0.12)**	1.13 (0.76, 1.68)	**0.17 (0.09, 0.3)**	1.01 (0.79, 1.28)
Calendar year (per year)	0.95 (0.9, 1.0)	0.96 (0.9, 1.02)	1.01 (0.94, 1.1)	1.04 (0.98, 1.11)	1 (0.96, 1.04)
Age (in years)					
<30 years	1 (Ref.)	1 (Ref.)	1 (Ref.)	1 (Ref.)	1 (Ref.)
30-39	0.93 (0.6, 1.44)	1.1 (0.55, 2.21)	0.93 (0.39, 2.19)	1.07 (0.59, 1.94)	1.76 (0.92, 3.35)
40-49	0.82 (0.51, 1.31)	1.41 (0.67, 2.93)	1.72 (0.74, 4)	0.89 (0.48, 1.65)	**2.18 (1.15, 4.14)**
50-59	0.76 (0.44, 1.32)	1.55 (0.72, 3.33)	2.4 (0.99, 5.81)	0.85 (0.43, 1.67)	**3.25 (1.69, 6.25)**
>60	0.94 (0.47, 1.85)	**3.13 (1.41, 6.97)**	**5.27 (2.14, 12.93)**	1.19 (0.42, 3.35)	**6.55 (3.32, 12.92)**
Sex: Female (vs. Male)	1.26 (0.98, 1.62)	1.04 (0.74, 1.47)	0.94 (0.65, 1.35)	1.2 (0.85, 1.69)	0.66 (0.51, 0.85)
IDU: Yes (vs. No)	1.07 (0.77, 1.48)	**2.01 (1.29, 3.12)**	1.55 (0.97, 2.46)	1.08 (0.71, 1.64)	1.29 (0.93, 1.78)
CD4 cell count (cells/mm^3^)					
>500	1 (Ref.)	1 (Ref.)	1 (Ref.)	1 (Ref.)	1 (Ref.)
351-500	0.81 (0.55, 1.18)	1.48 (0.94, 2.33)	**2.00 (1.23, 3.24)**	1.79 (0.85, 3.75)	1.44 (0.99, 2.08)
201-350	**1.76 (1.25, 2.48)**	**2.3 (1.52, 3.46)**	**2.06 (1.22, 3.47)**	**3.82 (1.88, 7.76)**	**2.25 (1.6, 3.18)**
51-200	**2.8 (1.9, 4.11)**	**5.76 (3.58, 9.26)**	**4.64 (2.66, 8.12)**	**11.73 (5.9, 23.32)**	**6.33 (4.5, 8.89)**
≤50	**9.71 (6.07, 15.52)**	**13.6 (7.55, 24.49)**	**15.9 (8.13, 31.28)**	**29.59 (13.83, 63.28)**	**27.32 (18.44, 40.47)**
HIV viral load (copies/mL)					
≤400	1 (Ref.)	1 (Ref.)	1 (Ref.)	1 (Ref.)	1 (Ref.)
401-3000	1.38 (0.94, 2.02)	1.51 (0.98, 2.33)	0.91 (0.52, 1.59)	**2.22 (1.11, 4.48)**	0.91 (0.64, 1.31)
3001-10000	**1.65 (1.07, 2.53)**	**1.91 (1.23, 2.97)**	1.13 (0.6, 2.1)	**3.09 (1.52, 6.31)**	1.03 (0.68, 1.56)
10001-100000	**2.52 (1.83, 3.46)**	**1.63 (1.11, 2.38)**	1.18 (0.77, 1.8)	**4.88 (2.9, 8.21)**	1.08 (0.81, 1.44)
>100000	**3.13 (2.1, 4.65)**	**2.49 (1.59, 3.9)**	1.15 (0.67, 1.99)	**8.03 (4.51, 14.29)**	**1.49 (1.07, 2.09)**
≤1 year since HIV + test: Yes (vs. No)	**2.72 (1.93, 3.83)**	1.17 (0.67, 2.03)	1.67 (0.96, 2.93)	**4.51 (3.14, 6.47)**	0.85 (0.53, 1.37)
On cART: Yes (vs. No)	**2.45 (1.3, 4.6)**	**8.3 (1.95, 35.33)**	**3.86 (1.17, 12.8)**	**7.62 (1.75, 33.26)**	0.85 (0.5, 1.47)
Hepatitis B positive (vs. not)	0.98 (0.61, 1.59)	1.07 (0.6, 1.91)	1.05 (0.61, 1.8)	1.31 (0.73, 2.37)	**1.96 (1.44, 2.66)**
Hepatitis C positive (vs. not)	1.11 (0.82, 1.51)	1.02 (0.65, 1.58)	1.31 (0.89, 1.94)	1.26 (0.85, 1.87)	**1.79 (1.34, 2.38)**

Bold indicates statistically significant results.

*IDU* injection drug use. *cART* combination antiretroviral therapy.

The incidence of non-AIDS events was increased for those with older age and who reported injection drug use. The incidence of non-AIDS events was also significantly higher for those with less than 1 year since first HIV positive test, those who used cART, and those with hepatitis C co-infection. Of note is the fact that, even after adjusting for confounders, IPEC still showed a lower incidence of non-AIDS events when compared to Aquitaine.

Age ≥ 60 years was associated with a higher incidence rate of several specific non-AIDS events categories, including bacterial, hepatic, neurological, and cardiovascular. Age ≥ 60 years showed a particularly strong association with cardiovascular disease (adjusted incidence rate ratio 5.27 95% CI: 2.14-12.93, Table 5). Injection drug use was associated with a higher incidence of psychiatric and neurological events. One year or less since first HIV positive test was significantly positively associated with the incidence of parasitic diseases. Hepatitis C co-infection was significantly associated with an increased incidence of hepatic diseases (adjusted incidence rate ratio 5.18 95% CI: 3.29-8.14).

Mortality was significantly higher with increasing age, decreasing immunity, increased plasma HIV RNA, and the presence of hepatitis B or C co-infection (Table 5).

## Discussion

We report here a unique analysis of multi-morbidity patterns among HIV-infected individuals followed in two geographically contrasting settings (Europe and South America) with universal access to treatment and care in the cART era. Over a 9-year period (2000–2008), ageing, decreased representation of IDUs, and improved immunological profile were observed in both cohorts largely exposed to cART. The rate of severe morbid events decreased by one-third over the period and, in 2008, only one out of five events was AIDS-related; non-AIDS events were always more frequent than AIDS events. Immunodeficiency was associated with a higher incidence of AIDS-related and all types of non-AIDS-related events. Our observations suggest that long-term restoration and preservation of high CD4 cell counts as a result of cART access is highly and durably effective in preventing AIDS as well as non-AIDS severe morbid events.

In this collaboration, sizeable efforts were allocated for the collection, documentation and uniform classification and coding of morbid events leading to hospitalizations. There is indeed a lack of published studies on the long-term causes of severe morbidity leading to hospitalization in HIV-infected patients, although such information is highly valuable in identifying priorities for case management and improvement of the quality of life of subjects living with a chronic disease requiring life-long treatment. Our study population combined two hospital-based cohorts that mainly differed by environment and only modestly by access to care. In this context, non-AIDS related conditions were the most common cause of hospitalizations throughout the study period.

The decreasing incidence rate of AIDS-related events over time confirms the improvements in HIV care as this finding is accompanied by a higher percentage of individuals with CD4 cell counts >500 cells/mm^3^ and undetectable plasma HIV RNAs. In fact, even beyond severe immunodeficiency, individuals with CD4 cell counts between 350–500 cells/mm^3^ were found to have a three times higher incidence rate of AIDS events than those with CD4 cell counts >500 cells/mm^3^. In addition, although the comparison of the incidence rate of AIDS events in Aquitaine and IPEC initially suggested that AIDS events occurred at much higher frequency in the latter, after adjustments for other characteristics (immunological and virological profile as well as time since first HIV positive test), this finding did not hold, suggesting that environment is not a primary determinant of the occurrence of AIDS severe diseases in a population highly exposed to effective antiretroviral combinations [16]. Universal access to cART in both settings as well as more potent and user-friendly cART regimens readily available along the study period [17-20] have indeed contributed to these findings.

Concerning mortality, the results paralleled the ones described above for AIDS-related events. Determinants of higher mortality rates were immunodeficiency, high plasma HIV RNA, ageing and co-infection with hepatitis B or C, rather than environment. Indeed, these findings corroborate a previous analysis that showed similar rates of early mortality between developed and resource-limited settings when adjusted for immunological and virological factors [13].

The relative higher incidence of non-AIDS-related events when compared to AIDS-related events throughout the study period confirms the recent findings of the significant burden of non-AIDS events among those living with HIV with a shift from AIDS to non-AIDS severe morbidity for the period 2000 to 2004 [10]. A recent study from the Swiss cohort also showed that non-AIDS-related events outnumbered HIV-related events in that setting in the years 2008 through 2010, and that non-AIDS-related events were associated with older age, similarly to our results [21]. Our adjusted analysis showed a lower incidence of non-AIDS events in the IPEC cohort even after taking into account possible confounding factors. An explanation for this finding might be differences in indication for hospitalization in the two settings. Although the overall rates of non-AIDS-related events have significantly decreased between 2000 and 2008, the incidence of bacterial, cardiovascular, hepatic and hematological events remained stable over time. Prevention programs targeting tobacco use cessation, vaccination against influenza and pneumococcal diseases can greatly impact the most frequent non-AIDS severe morbid event category, i.e. bacterial infections. As for non-AIDS defining malignancies, existing screening policies such as anal cancer screening could be applied and additional screening policies, for example for lung cancer, should be evaluated.

Bacterial events were by far the most frequent category with no decreasing trend over the years. A stabilization was also identified in the most recent analysis of North American cohorts [3] where a classification system similar to the one used in the present study was employed. Differently from HOPS, NHS, or prior HIVRN [8,22], the study by Berry et al. [3] and the present study classified infections (bacterial, viral or parasitic) as hierarchically higher than end-organ categories because antimicrobials are the primary therapy and many cases require specialized case management. In a combined analysis of two cohorts from Côte d’Ivoire, bacterial events were also among the most frequent events that led to hospitalization (23% of all hospitalizations [11]). In the latter study, though differently from the present analysis, only a minority of patients were receiving cART [11]. Hence, bacterial events do not seem to be impacted by the increase in cART use and represent an important disease burden especially for septicemia and septic shock with important costs for drugs and intensive care units [23]. The high burden of bacterial infections in our study population indicates an urgent need for additional approaches concerning preventable bacterial infections, such as expansion of vaccination for influenza and pneumococcal disease.

Over the years, our study population aged significantly, more likely as a result of patients living longer with HIV/AIDS than of older patients reaching care. As a result, the incidence rate of severe morbid events associated with increased age, such as non-AIDS-defining malignancies, cardiovascular diseases and other end-organ diseases could be expected to increase with time. Although improved management of traditional cardiovascular risk factors and the use of more lipid friendlier ART regimens may have a favorable impact on the incidence of cardiovascular events, the French hospital database showed that HIV replication and immune status are independent predictors of myocardial infarction in HIV-infected individuals [24]. Further investigations should look for optimal monitoring strategies for non-AIDS co-morbidities in individuals on and off cART both in high- and middle/low-income countries, especially among those aged 50 and above.

Hepatic failure secondary to viral hepatitis was the most frequent hepatic event in our analysis. Other studies have demonstrated the increasing impact of chronic viral hepatitis and liver disease on hospital admission rates [22,25-27]. Prevention of hepatitis co-infection, timely treatment of chronic viral hepatitis in HIV co-infected patients, and careful monitoring of treatment related hepatotoxicity are important measures to reduce liver-related complications and subsequent hospitalizations.

In the present analysis severe morbidity requiring hospitalization due to non-AIDS malignancies did not show an increasing trend over time. Berry et al., in a contemporary analysis (2001–2008), were also unable to find an increase in hospitalizations for non-AIDS malignancies [3]. In contrast, it was recently shown that the relative frequency of deaths due to non-AIDS malignancies have increased in France [28]. In addition, large epidemiological studies has shown an increased incidence of non-AIDS malignancies in HIV-infected groups, two-fold higher than that found in the general population [29,30]. However, these latter studies included data up to 2002 and therefore did not take into account the long-term benefit of cART. Other recent studies of cancer incidence have reported increases in specific cancers (such as anal cancer and Hodgkin Disease) but no clear increase in overall non-AIDS malignancies since 2001 [31]. In our study, malignant neoplasm of the lung was the most frequent diagnosis among the non-AIDS malignancies category. As shown by several studies, the incidence of this malignancy is increased with HIV infection and even more so among patients with AIDS compared to demographically similar populations [32-34]. It has been suggested that immunodeficiency-induced recurrent pneumonia and its associated inflammation may contribute to lung carcinogenesis in HIV infected individuals [35]. Given the lack of effective treatment options and as HIV-infected individuals live longer in the era of cART, it is expected that smoking will increasingly manifest its oncogenic potential and that lung cancer may become an increasingly important cause of death. Tobacco cessation programs already in place for the ANRS CO3 Aquitaine Cohort, as well as in other settings, can help reduce lung cancer incidence in this scenario [36,37].

Among the specific non-AIDS categories, our study shows that psychiatric and neurologic diseases impose a significant burden. Although the incidence rate of psychiatric disease showed decreasing trends over the study period, it was the second most frequent cause of severe morbidity which shows the need for access to outpatient psychiatric care and coverage of psychotropic medicines, as well as careful monitoring of interactions between cART, in particular of efavirenz [38] and psychiatric medications. In our adjusted analyses we found that IDU was significantly associated with a higher overall rate of non-AIDS events, being most likely driven by psychiatric and neurologic severe morbidity. In addition, ageing was also found to be associated with neurologic diseases. This finding, when coupled with the most frequent diagnoses reported in the neurologic category (headache and dizziness) suggests an increased rate of hospital admissions for investigational purposes but without a concrete discharge diagnosis.

The present study has important strengths and limitations. This is the first study to compare causes of severe morbidity from the Northern and Southern hemispheres. In this study, a severe morbid event was defined as any event associated with a hospitalization for at least 48 hours. Thus, the studied events were on the same level of severity. In addition, both cohorts have reference hospitals to which patients refer to for hospitalizations, thus substantially decreasing the chances of missed hospitalizations. On the other hand, by definition, severe morbid events requiring hospitalization of less than 48 hours were excluded and thus it is possible that a fraction of severe morbid events were not represented in this study. Differently from Crum-Cianflone et al. [22], where each hospitalization was placed into a single category, we captured all diagnoses associated with a hospitalization thus allowing for a more thorough description of the causes and trends of severe morbid events. The present study did not explore the impact of different cART regimens and further studies are needed to evaluate the impact of different treatments (in particular of efavirenz-based treatments) on the overall and specific non-AIDS severe morbidities [38]. The present study sought to harmonize hospital discharge diagnoses of two distinct hospital-based cohorts into comprehensive categories but decisions to group specific diagnoses in one or another category were made arbitrarily and thus could have influenced the results. As we move forward from evaluating mortality alone to include morbidity, data harmonization will become an increasing challenge. Finally, information on the presence of risk factors of severe morbidity, such as tobacco use and alcohol consumption, on important comorbidities as diabetes and hypertension, on the use of preventive interventions such as vaccinations, and on the use of treatment for other comorbidities such hepatitis B/C infections were not taken into account in this study as data was not systematically available. Efforts should be made in all cohorts of HIV-infected individuals to improve the information collected concerning these determinants.

Overall, our study shows that lower CD4 cell counts led to a higher incidence rate of both AIDS-related and non-AIDS-related events. Indeed, only 15% of the events occurred in the CD4 stratum >500 cells/mm^3^. Several studies have also noted the relationship between lower CD4 counts and severe morbidity [8,22,26]. Higher plasma HIV RNA and shorter time since first HIV positive test were significantly associated with a higher incidence rate of severe morbidity. Late diagnosis is still a major public health issue both in France and in Brazil [39,40]. Our results emphasize the critical need for earlier HIV diagnosis, linkage to care and prompt cART initiation to maintain robust CD4 counts to further reduce morbidity and mortality. Non-AIDS severe morbidity was associated with older age. Increasing age was also associated with increased hospital admissions, especially for non-AIDS causes in other studies [22]. Ageing of the HIV population may be contributing to the high hospitalization rates, a trend that will likely persist in the upcoming years. Poly-pathology, which increases with ageing, may also play a role [41,42].

## Conclusions

In conclusion, our study shows improvements up until 2008 both in South Western France and the region of Rio de Janeiro and allows the identification of priorities for improving the quality of life of patients, beyond specific HIV treatment. Cohorts should increase efforts to record and analyze non-AIDS severe morbidity both in the North and the South, to better reflect the disease burden of a chronic disease requiring life-long treatment and grasp the future epidemiological trends as equally and accurately as possible.

## Appendix

The ANRS CO3 Aquitaine Cohort study group:

**Principal investigator**: Pr F. Dabis.

**Scientific committee**: Prs F. Bonnet, D. Breilh, F. Dabis, M. Dupon, G. Chêne, H. Fleury, D. Malvy, P. Mercié, I. Pellegrin, P. Morlat, D. Neau, JL. Pellegrin, R. Thiébaut, Drs S. Bouchet, V. Gaborieau, D. Lacoste, S. Tchamgoué, L. Wittkop.

**Composition of the GECSA**: Epidemiology and biostatistics: Prs G. Chêne, F. Dabis, R. Thiébaut, Drs M. Bruyand, S. Lawson-Ayayi, L. Wittkop.

**Clinical and biological hospital units**: Bordeaux University Hospital: Pr P. Morlat (Pr F. Bonnet, Drs N. Bernard, M. Hessamfar, D. Lacoste, MA. Vandenhende), Pr M. Dupon (Drs FA. Dauchy, H. Dutronc), Pr P. Mercié (Drs P. Duffau, J. Roger Schmeltz), Pr D. Malvy (Drs T. Pistone, MC Receveur), Pr D. Neau (Drs C. Cazanave, A. Ochoa, MO. Vareil), Pr JL. Pellegrin (Pr JF. Viallard, Drs C. Greib, E. Lazaro), Pr H. Fleury (Pr ME. Lafon, Drs S. Reigadas, P. Trimoulet), Pr D. Breilh, Pr M. Molimard (Drs S. Bouchet, K. Titier), Pr JF. Moreau (Dr I. Pellegrin), Drs F. Haramburu, G. Miremont-Salamé. Arcachon Hospital: Dr A. Dupont. Dax Hospital: Dr Y. Gerard (Drs L. Caunègre, K. André). Bayonne Hospital: Dr F. Bonnal (Drs S. Farbos, MC. Gemain). Libourne Hospital: Dr J. Ceccaldi (Drs O. Caubet, S. Tchamgoué). Mont-de-Marsan Hospital: Dr S. De Witte (Dr C. Courtault). Pau Hospital: Drs E. Monlun (Dr V. Gaborieau). Périgueux Hospital: Dr P. Lataste (Dr JP. Meraud). Villeneuve-sur-Lot Hospital: Dr I. Chossat.

**Permanent team**: MJ. Blaizeau, M. Bruyand, V. Conte, M. Decoin, J. Delaune, S. Delveaux, F. Diarra, C. D’Ivernois, A. Frosch, S. Geffard, C. Hanappier, S. Lawson-Ayayi, E. Lenaud, O. Leleux, F. Le Marec, J. Leray, I. Louis, G. Palmer, A. Pougetoux, X. Sicard, D. Touchard B. Uwamaliya-Nziyumvira.

## Competing interests

The authors declare that they have no competing interests.

## Authors’ contributions

PML conceived of the study and participated in its design and coordination, performed the statistical analysis and drafted the manuscript. SR, FB, RIM, MH, DPC, CG, CC attended the patients, participated in the data collection and validation and helped to draft the manuscript. MB, VGV, FD, BG, and GC conceived of the study, and participated in its design and coordination and helped to draft the manuscript. All authors have given final approval of the version to be published and agree to be accountable for all aspects of the work in ensuring that questions related to the accuracy or integrity of any part of the work are appropriately investigated and resolved.

## Pre-publication history

The pre-publication history for this paper can be accessed here:

http://www.biomedcentral.com/1471-2334/14/278/prepub

## Supplementary Material

Additional file 1: Table S1 Demographic and clinic characteristics (number [percent]) of the ANRS CO3 Aquitaine Cohort study population by year. **Table S2.** Demographic and clinic characteristics (number [percent]) of the IPEC Cohort study population by year. **Table S3.** Annual incidence rates per 100 person-years of non-AIDS events ranked from most to least frequent for the ANRS CO3Aquitaine Cohort. **Table S4.** Annual incidence rates per 100 person-years of non-AIDS events ranked from most to least frequent for the IPEC Cohort. **Table S5.** Three most frequent diagnoses within the AIDS and specific non-AIDS severe morbid events categories in the ANRS CO3 Aquitaine Cohort. **Table S6.** Three most frequent diagnoses within the AIDS and specific non-AIDS severe morbid events categories in the IPEC Cohort. Click here for file
