# Supplementary material for: AIDS and non-AIDS severe morbidity associated with hospitalizations among HIV-infected patients in two regions with universal access to care and antiretroviral therapy, France and Brazil, 2000–2008: hospital-based cohort studies
Source: BMC Infect Dis. 2014 May 21;14:278. doi: 10.1186/1471-2334-14-278 (PMC4032588; doi:10.1186/1471-2334-14-278)
Supplement: Additional file 1: Table S1 — Demographic and clinic characteristics (number [percent]) of the ANRS CO3 Aquitaine Cohort study population by year. Table S2. Demographic and clinic characteristics (number [percent]) of the IPEC Cohort study population by year. Table S3. Annual incidence rates per 100 person-years of non-AIDS events ranked from most to least frequent for the ANRS CO3Aquitaine Cohort. Table S4. Annual incidence rates per 100 person-years of non-AIDS events ranked from most to least frequent for the IPEC Cohort. Table S5. Three most frequent diagnoses within the AIDS and specific non-AIDS severe morbid events categories in the ANRS CO3 Aquitaine Cohort. Table S6. Three most frequent diagnoses within the AIDS and specific non-AIDS severe morbid events categories in the IPEC Cohort. [file 1471-2334-14-278-S1.docx]

**Additional file 1**

**Table S1:** Demographic and clinic characteristics (number [percent]) of the ANRS CO3 Aquitaine Cohort study population by year.

|  | **2000** | **2001** | **2002** | **2003** | **2004** | **2005** | **2006** | **2007** | **2008** |
| --- | --- | --- | --- | --- | --- | --- | --- | --- | --- |
| Number of patients | 3079 | 3167 | 3274 | 3364 | 3558 | 3740 | 3813 | 3858 | 3819 |
| Age in years ^a^ |  |  |  |  |  |  |  |  |  |
| < 30 | 275 (8.9) | 242 (7.6) | 208 (6.4) | 192 (5.7) | 183 (5.1) | 212 (5.7) | 202 (5.3) | 189 (4.9) | 178 (4.7) |
| 30-39 | 1490 (48.4) | 1397 (44.1) | 1340 (40.9) | 1234 (36.7) | 1155 (32.5) | 1031 (27.6) | 914 (24) | 837 (21.7) | 735 (19.2) |
| 40-49 | 884 (28.7) | 1019 (32.2) | 1154 (35.2) | 1291 (38.4) | 1478 (41.5) | 1646 (44) | 1760 (46.2) | 1801 (46.7) | 1770 (46.3) |
| 50-59 | 285 (9.3) | 351 (11.1) | 412 (12.6) | 462 (13.7) | 520 (14.6) | 588 (15.7) | 645 (16.9) | 688 (17.8) | 741 (19.4) |
| >= 60 | 145 (4.7) | 158 (5) | 160 (4.9) | 185 (5.5) | 222 (6.2) | 263 (7) | 292 (7.7) | 343 (8.9) | 395 (10.3) |
| Gender |  |  |  |  |  |  |  |  |  |
| Male | 2255 (73.2) | 2307 (72.8) | 2392 (73.1) | 2453 (72.9) | 2594 (72.9) | 2714 (72.6) | 2751 (72.1) | 2784 (72.2) | 2768 (72.5) |
| Mode of HIV transmission ^b^ | | |  |  |  |  |  |  |  |
| Heterosexual | 702 (22.8) | 759 (24) | 805 (24.6) | 861 (25.6) | 953 (26.8) | 1060 (28.3) | 1101 (28.9) | 1148 (29.8) | 1157 (30.3) |
| MSM | 1122 (36.4) | 1155 (36.5) | 1220 (37.3) | 1283 (38.1) | 1368 (38.4) | 1451 (38.8) | 1491 (39.1) | 1520 (39.4) | 1528 (40) |
| IDU | 980 (31.8) | 963 (30.4) | 953 (29.1) | 921 (27.4) | 933 (26.2) | 915 (24.5) | 899 (23.6) | 870 (22.6) | 820 (21.5) |
| Other | 116 (3.8) | 120 (3.8) | 120 (3.7) | 123 (3.7) | 124 (3.5) | 124 (3.3) | 127 (3.3) | 121 (3.1) | 122 (3.2) |
| Unknown | 159 (5.2) | 170 (5.4) | 176 (5.4) | 176 (5.2) | 180 (5.1) | 190 (5.1) | 195 (5.1) | 199 (5.2) | 192 (5) |
| CD4 cell count ^c^ |  |  |  |  |  |  |  |  |  |
| > 500 | 1077 (39.7) | 1147 (41.3) | 1293 (43.6) | 1350 (45) | 1328 (41.8) | 1485 (43.5) | 1648 (47.8) | 1714 (48.4) | 1845 (51.3) |
| 351-500 | 637 (23.5) | 676 (24.3) | 717 (24.2) | 711 (23.7) | 803 (25.3) | 859 (25.1) | 861 (25) | 912 (25.8) | 924 (25.7) |
| 201-350 | 605 (22.3) | 588 (21.2) | 580 (19.5) | 609 (20.3) | 697 (21.9) | 699 (20.5) | 624 (18.1) | 598 (16.9) | 555 (15.4) |
| 51-200 | 317 (11.7) | 289 (10.4) | 301 (10.1) | 273 (9.1) | 284 (8.9) | 304 (8.9) | 253 (7.3) | 269 (7.6) | 231 (6.4) |
| <= 50 | 75 (2.8) | 79 (2.8) | 77 (2.6) | 58 (1.9) | 67 (2.1) | 69 (2) | 62 (1.8) | 48 (1.4) | 38 (1.1) |
| Plasma HIV RNA ^c^ | |  |  |  |  |  |  |  |  |
| <= 400 | 1008 (37.2) | 1106 (39.9) | 1311 (44.6) | 1475 (49.3) | 1635 (51.5) | 1945 (57.2) | 2139 (62.3) | 2417 (68.6) | 2735 (76.2) |
| 401-3000 | 608 (22.4) | 632 (22.8) | 548 (18.6) | 459 (15.4) | 462 (14.6) | 417 (12.3) | 379 (11) | 323 (9.2) | 282 (7.9) |
| 3001-10000 | 357 (13.2) | 342 (12.3) | 333 (11.3) | 305 (10.2) | 275 (8.7) | 267 (7.8) | 217 (6.3) | 180 (5.1) | 144 (4) |
| 10001-100000 | 542 (20) | 519 (18.7) | 556 (18.9) | 579 (19.4) | 614 (19.4) | 568 (16.7) | 479 (13.9) | 451 (12.8) | 323 (9) |
| > 100000 | 195 (7.2) | 174 (6.3) | 193 (6.6) | 172 (5.8) | 186 (5.9) | 205 (6) | 221 (6.4) | 150 (4.3) | 104 (2.9) |

**(CONT. Table S1)**

|  | **2000** | **2001** | **2002** | **2003** | **2004** | **2005** | **2006** | **2007** | **2008** |
| --- | --- | --- | --- | --- | --- | --- | --- | --- | --- |
| ≤ 1 year since first HIV+ test ^d^ | | | |  |  |  |  |  |  |
| Yes | 250 (8.1) | 176 (5.6) | 206 (6.3) | 212 (6.3) | 206 (5.8) | 249 (6.7) | 214 (5.6) | 189 (4.9) | 158 (4.1) |
| On cART ^e^ | |  |  |  |  |  |  |  |  |
| Yes | 2833 (92) | 2913 (92) | 3003 (91.7) | 3073 (91.3) | 3248 (91.3) | 3417 (91.4) | 3483 (91.3) | 3560 (92.3) | 3583 (93.8) |
| Hepatitis B ^f^ |  |  |  |  |  |  |  |  |  |
| Yes | 193 (6.8) | 207 (7.1) | 217 (7.2) | 220 (7) | 231 (7) | 241 (6.9) | 256 (7.2) | 260 (7.3) | 261 (7.4) |
| Hepatitis C, N (%) |  |  |  |  |  |  |  |  |  |
| Yes | 873 (31.1) | 883 (30.4) | 915 (30.4) | 906 (29.1) | 901 (27.5) | 880 (25.5) | 873 (24.9) | 850 (24) | 832 (23.7) |

^a^ Age from birth was updated yearly for each participant

^b^ Presumed mode of HIV exposure was classified as men who have sex with men (MSM), injection drug use (IDU), heterosexual transmission, other, and unknown

^c^ Yearly CD4 cell counts and plasma HIV RNAs were defined as the mean value of all measurements taken in a specific calendar year

^d^ Time since first HIV+ test was calculated for each calendar year

^e^ Combination antiretroviral therapy (cART) was defined as two nucleoside reverse transcriptase inhibitors plus either one non-nucleoside reverse transcriptase inhibitor or one protease-inhibitor (boosted or not)

^f^ Hepatitis B infection was defined by the presence of hepatitis B surface antigen (HBsAg)

^g^ Hepatitis C infection was defined by the presence of hepatitis C antibodies

**Table S2:** Demographic and clinic characteristics (number [percent]) of the IPEC Cohort study population by year.

|  | **2000** | **2001** | **2002** | **2003** | **2004** | **2005** | **2006** | **2007** | **2008** |
| --- | --- | --- | --- | --- | --- | --- | --- | --- | --- |
| Number of patients | 832 | 998 | 1101 | 1204 | 1340 | 1527 | 1808 | 2058 | 2325 |
| Age in years ^a^ |  |  |  |  |  |  |  |  |  |
| < 30 | 155 (18.6) | 177 (17.7) | 156 (14.2) | 144 (12) | 142 (10.6) | 172 (11.3) | 224 (12.4) | 267 (13) | 305 (13.1) |
| 30-39 | 340 (40.9) | 411 (41.2) | 447 (40.6) | 456 (37.9) | 459 (34.3) | 532 (34.8) | 587 (32.5) | 657 (31.9) | 727 (31.3) |
| 40-49 | 243 (29.2) | 297 (29.8) | 351 (31.9) | 424 (35.2) | 509 (38) | 537 (35.2) | 662 (36.6) | 746 (36.2) | 833 (35.8) |
| 50-59 | 74 (8.9) | 84 (8.4) | 106 (9.6) | 135 (11.2) | 180 (13.4) | 223 (14.6) | 260 (14.4) | 294 (14.3) | 352 (15.1) |
| >= 60 | 20 (2.4) | 29 (2.9) | 41 (3.7) | 45 (3.7) | 50 (3.7) | 63 (4.1) | 75 (4.1) | 94 (4.6) | 108 (4.6) |
| Gender, N (%) |  |  |  |  |  |  |  |  |  |
| Male | 470 (56.5) | 572 (57.3) | 647 (58.8) | 722 (60) | 824 (61.5) | 965 (63.2) | 1147 (63.4) | 1312 (63.8) | 1488 (64) |
| Mode of HIV transmission ^b^ | | |  |  |  |  |  |  |  |
| Heterosexual | 427 (51.3) | 515 (51.6) | 566 (51.4) | 603 (50.1) | 660 (49.3) | 738 (48.3) | 893 (49.4) | 1028 (50) | 1171 (50.4) |
| MSM | 261 (31.4) | 316 (31.7) | 355 (32.2) | 399 (33.1) | 449 (33.5) | 512 (33.5) | 611 (33.8) | 701 (34.1) | 792 (34.1) |
| IDU | 31 (3.7) | 33 (3.3) | 31 (2.8) | 34 (2.8) | 32 (2.4) | 32 (2.1) | 34 (1.9) | 39 (1.9) | 38 (1.6) |
| Other | 29 (3.5) | 30 (3) | 31 (2.8) | 32 (2.7) | 31 (2.3) | 33 (2.2) | 38 (2.1) | 40 (1.9) | 44 (1.9) |
| Unknown | 84 (10.1) | 104 (10.4) | 118 (10.7) | 136 (11.3) | 168 (12.5) | 212 (13.9) | 232 (12.8) | 250 (12.1) | 280 (12) |
| CD4 cell count ^c^ |  |  |  |  |  |  |  |  |  |
| > 500 | 229 (32.7) | 265 (33.2) | 287 (32.5) | 296 (31.9) | 440 (37.6) | 511 (38) | 626 (38.6) | 743 (39.5) | 875 (40.3) |
| 351-500 | 150 (21.4) | 190 (23.8) | 203 (23) | 213 (22.9) | 272 (23.3) | 308 (22.9) | 364 (22.4) | 463 (24.6) | 529 (24.4) |
| 201-350 | 172 (24.6) | 195 (24.4) | 215 (24.3) | 243 (26.2) | 234 (20) | 264 (19.6) | 373 (23) | 387 (20.6) | 462 (21.3) |
| 51-200 | 110 (15.7) | 121 (15.1) | 148 (16.8) | 141 (15.2) | 162 (13.9) | 198 (14.7) | 206 (12.7) | 232 (12.3) | 248 (11.4) |
| <= 50 | 39 (5.6) | 28 (3.5) | 30 (3.4) | 36 (3.9) | 61 (5.2) | 63 (4.7) | 53 (3.3) | 55 (2.9) | 55 (2.5) |
| Plasma HIV RNA ^c^ | |  |  |  |  |  |  |  |  |
| <= 400 | 143 (20.5) | 218 (27.9) | 317 (34.9) | 384 (40.8) | 465 (41.2) | 569 (43.8) | 769 (49.9) | 931 (52.2) | 1163 (55) |
| 401-3000 | 141 (20.2) | 163 (20.9) | 141 (15.5) | 139 (14.8) | 151 (13.4) | 187 (14.4) | 178 (11.6) | 188 (10.5) | 236 (11.2) |
| 3001-10000 | 118 (16.9) | 109 (14) | 112 (12.3) | 104 (11) | 118 (10.5) | 129 (9.9) | 128 (8.3) | 174 (9.7) | 174 (8.2) |
| 10001-100000 | 183 (26.2) | 197 (25.3) | 225 (24.8) | 210 (22.3) | 257 (22.8) | 294 (22.6) | 308 (20) | 354 (19.8) | 398 (18.8) |
| > 100000 | 113 (16.2) | 93 (11.9) | 113 (12.4) | 105 (11.1) | 137 (12.1) | 120 (9.2) | 157 (10.2) | 138 (7.7) | 142 (6.7) |

**(CONT. Table S2)**

|  | **2000** | **2001** | **2002** | **2003** | **2004** | **2005** | **2006** | **2007** | **2008** |
| --- | --- | --- | --- | --- | --- | --- | --- | --- | --- |
| ≤ 1 year since first HIV+ test ^d^ | | | |  |  |  |  |  |  |
| Yes | 189 (22.7) | 174 (17.4) | 131 (11.9) | 104 (8.6) | 110 (8.2) | 172 (11.3) | 279 (15.4) | 307 (14.9) | 343 (14.8) |
| On cART ^e^ | |  |  |  |  |  |  |  |  |
| Yes | 673 (80.9) | 811 (81.3) | 929 (84.4) | 1028 (85.4) | 1145 (85.4) | 1267 (83) | 1430 (79.1) | 1637 (79.5) | 1925 (82.8) |
| Hepatitis B ^f^ |  |  |  |  |  |  |  |  |  |
| Yes | 22 (3) | 28 (3.2) | 32 (3.2) | 38 (3.5) | 49 (4) | 64 (4.6) | 75 (4.5) | 88 (4.8) | 106 (5.3) |
| Hepatitis C ^g^ |  |  |  |  |  |  |  |  |  |
| Yes | 76 (10.3) | 85 (9.5) | 98 (9.7) | 116 (10.4) | 126 (10) | 128 (8.7) | 145 (8.3) | 149 (7.6) | 146 (6.8) |

^a^ Age from birth was updated yearly for each participant

^b^ Presumed mode of HIV exposure was classified as men who have sex with men (MSM), injection drug use (IDU), heterosexual transmission, other, and unknown

^c^ Yearly CD4 cell counts and plasma HIV RNAs were defined as the mean value of all measurements taken in a specific calendar year

^d^ Time since first HIV+ test was calculated for each calendar year

^e^ Combination antiretroviral therapy (cART) was defined as two nucleoside reverse transcriptase inhibitors plus either one non-nucleoside reverse transcriptase inhibitor or one protease-inhibitor (boosted or not)

^f^ Hepatitis B infection was defined by the presence of hepatitis B surface antigen (HBsAg)

^g^ Hepatitis C infection was defined by the presence of hepatitis C antibodies

**Table S3:** Annual incidence rates per 100 person-years of non-AIDS events ranked from most to least frequent for the ANRS CO3Aquitaine Cohort.

|  |  | **2000** | **2001** | **2002** | **2003** | **2004** | **2005** | **2006** | **2007** | **2008** | **P-value** | **Total # of events** |
| --- | --- | --- | --- | --- | --- | --- | --- | --- | --- | --- | --- | --- |
| **1** | **Bacterial** | 3.93 | 2.98 | 2.94 | 2.37 | 2.87 | 2.42 | 2.90 | 3.03 | 2.12 | 0.03 | 842 |
| **2** | **Psychiatric** | 2.75 | 2.14 | 1.78 | 1.66 | 2.55 | 2.08 | 2.43 | 2.04 | 1.81 | 0.35 | 637 |
| **3** | **Hepatic** | 1.17 | 1.91 | 1.16 | 1.09 | 1.38 | 1.82 | 1.60 | 1.73 | 1.45 | 0.16 | 445 |
| **4** | **Neurological** | 1.72 | 2.41 | 1.42 | 1.75 | 1.20 | 1.42 | 1.05 | 0.91 | 1.51 | 0.02 | 437 |
| **5** | **Hematological** | 0.96 | 1.94 | 1.42 | 1.37 | 1.50 | 1.17 | 1.16 | 1.35 | 1.53 | 0.84 | 411 |
| **6** | **Viral** | 2.41 | 1.31 | 1.36 | 1.12 | 1.11 | 0.97 | 0.77 | 0.85 | 1.09 | < 0.01 | 356 |
| **7** | **Digestive** | 1.86 | 1.64 | 1.00 | 1.25 | 1.02 | 1.14 | 1.22 | 0.83 | 0.92 | < 0.01 | 355 |
| **8** | **Parasitic** | 1.27 | 0.94 | 0.74 | 1.28 | 0.99 | 0.54 | 0.55 | 0.83 | 0.59 | < 0.01 | 252 |
| **9** | **Cardiovascular** | 0.62 | 1.17 | 0.74 | 0.87 | 0.69 | 0.37 | 0.86 | 0.85 | 1.26 | 0.40 | 247 |
| **10** | **Signs/symptoms** | 1.14 | 0.87 | 0.26 | 0.50 | 0.54 | 0.77 | 0.58 | 1.02 | 0.36 | 0.35 | 199 |
| **11** | **Rheumatological** | 0.48 | 0.47 | 0.45 | 0.28 | 0.27 | 0.54 | 0.66 | 0.94 | 0.61 | 0.03 | 159 |
| **12** | **Respiratory** | 0.69 | 0.40 | 0.65 | 0.47 | 0.18 | 0.28 | 0.36 | 0.69 | 0.59 | 0.88 | 142 |
| **13** | **Renal** | 0.55 | 0.30 | 0.49 | 0.16 | 0.39 | 0.40 | 0.47 | 0.74 | 0.64 | 0.09 | 139 |
| **14** | **Malignancies** | 0.38 | 0.54 | 0.19 | 0.69 | 0.12 | 0.23 | 0.33 | 0.55 | 0.67 | 0.34 | 123 |
| **15** | **Dermatological** | 0.59 | 0.47 | 0.39 | 0.56 | 0.33 | 0.28 | 0.30 | 0.30 | 0.22 | < 0.01 | 112 |
| **16** | **Endocrine** | 0.24 | 0.23 | 0.10 | 0.31 | 0.36 | 0.40 | 0.25 | 0.36 | 0.31 | 0.21 | 86 |
| **17** | **Other** | 0.52 | 0.44 | 0.32 | 0.31 | 0.18 | 0.17 | 0.08 | 0.28 | 0.14 | < 0.01 | 78 |
| **18** | **Ophtamological** | 0.10 | 0.50 | 0.03 | 0.03 | 0.12 | 0.03 | 0.11 | 0.14 | 0.17 | 0.36 | 40 |
| **19** | **Trauma** | 0.10 | 0.00 | 0.13 | 0.03 | 0.00 | 0.06 | 0.17 | 0.08 | 0.17 | 0.19 | 25 |
| **20** | **Gynecological** | 0.03 | 0.10 | 0.03 | 0.03 | 0.03 | 0.09 | 0.00 | 0.03 | 0.06 | 0.68 | 13 |
| **21** | **In situ neoplasia** | 0.03 | 0.00 | 0.00 | 0.03 | 0.00 | 0.00 | 0.00 | 0.00 | 0.00 | NA | 2 |

**Table S4:** Annual incidence rates per 100 person-years of non-AIDS events ranked from most to least frequent for the IPEC Cohort.

|  |  | **2000** | **2001** | **2002** | **2003** | **2004** | **2005** | **2006** | **2007** | **2008** | **P-value** | **Total # of events** |
| --- | --- | --- | --- | --- | --- | --- | --- | --- | --- | --- | --- | --- |
| **1** | **Bacterial** | 6.74 | 9.04 | 5.98 | 8.50 | 6.74 | 6.37 | 5.37 | 5.11 | 6.26 | 0.06 | 760 |
| **2** | **Cardiovascular** | 2.16 | 1.26 | 0.80 | 1.00 | 0.74 | 1.33 | 0.95 | 1.24 | 0.82 | 0.24 | 128 |
| **3** | **Hepatic** | 1.35 | 2.17 | 1.00 | 1.28 | 1.07 | 0.89 | 0.82 | 0.59 | 1.20 | 0.08 | 127 |
| **4** | **Viral** | 2.02 | 1.37 | 1.99 | 1.28 | 1.23 | 0.89 | 0.51 | 0.43 | 1.01 | < 0.01 | 125 |
| **5** | **Psychiatric** | 0.94 | 1.03 | 1.20 | 1.83 | 0.82 | 0.67 | 0.51 | 0.21 | 0.53 | < 0.01 | 90 |
| **6** | **Digestive** | 1.08 | 0.92 | 0.50 | 1.10 | 0.41 | 0.59 | 0.51 | 0.64 | 0.58 | 0.18 | 78 |
| **7** | **Endocrine** | 1.21 | 1.72 | 0.60 | 0.73 | 0.66 | 0.67 | 0.19 | 0.54 | 0.29 | < 0.01 | 74 |
| **8** | **Hematological** | 0.67 | 0.57 | 0.80 | 0.82 | 0.90 | 0.59 | 0.63 | 0.11 | 0.48 | 0.09 | 68 |
| **9** | **Renal** | 0.94 | 0.11 | 0.60 | 0.91 | 0.33 | 0.59 | 0.38 | 0.81 | 0.24 | 0.33 | 62 |
| **10** | **Parasitic** | 0.67 | 0.46 | 0.40 | 0.46 | 0.25 | 0.37 | 0.44 | 0.32 | 0.34 | 0.45 | 46 |
| **11** | **Dermatological** | 0.54 | 0.69 | 0.40 | 0.18 | 0.08 | 0.30 | 0.19 | 0.43 | 0.29 | 0.32 | 38 |
| **12** | **Respiratory** | 0.40 | 0.57 | 0.30 | 0.09 | 0.08 | 0.07 | 0.00 | 0.11 | 0.10 | 0.03 | 18 |
| **13** | **Signs/symptoms** | 0.00 | 0.00 | 0.20 | 0.09 | 0.00 | 0.15 | 0.06 | 0.16 | 0.34 | 0.05 | 16 |
| **14** | **Neurological** | 0.13 | 0.11 | 0.10 | 0.00 | 0.25 | 0.15 | 0.19 | 0.00 | 0.10 | 0.68 | 13 |
| **15** | **Malignancies** | 0.27 | 0.23 | 0.10 | 0.00 | 0.00 | 0.00 | 0.19 | 0.05 | 0.10 | 0.46 | 11 |
| **16** | **Other** | 0.13 | 0.00 | 0.10 | 0.00 | 0.00 | 0.07 | 0.00 | 0.05 | 0.05 | NA | 5 |
| **17** | **Rheumatological** | 0.13 | 0.11 | 0.10 | 0.00 | 0.08 | 0.00 | 0.00 | 0.00 | 0.00 | NA | 4 |
| **18** | **Trauma** | 0.00 | 0.00 | 0.00 | 0.00 | 0.00 | 0.00 | 0.06 | 0.00 | 0.00 | NA | 1 |
| **-** | **In situ neoplasia** | 0.00 | 0.00 | 0.00 | 0.00 | 0.00 | 0.00 | 0.00 | 0.00 | 0.00 | NA | 0 |
| **-** | **Gynecological** | 0.00 | 0.00 | 0.00 | 0.00 | 0.00 | 0.00 | 0.00 | 0.00 | 0.00 | NA | 0 |
| **-** | **Ophtamological** | 0.00 | 0.00 | 0.00 | 0.00 | 0.00 | 0.00 | 0.00 | 0.00 | 0.00 | NA | 0 |

**Table S5:** Three most frequent diagnoses within the AIDS and specific non-AIDS severe morbid events categories in the ANRS CO3 Aquitaine Cohort.

| **Diagnosis (ICD-10 code)** | **N/N (%)** |
| --- | --- |
| **AIDS** |  |
| Pneumocystosis (B59) | 123/863 (14.3) |
| Neurotoxoplasmosis (B58.2) | 98/863 (11.4) |
| Esophageal candidiasis (B37.2) | 85/863 (9.8) |
| **Non-AIDS** |  |
| **Bacterial** |  |
| Pneumonia, other specified infectious organisms (J16.8) | 127/842 (15.1) |
| Pneumonia, unspecified (J18.9) | 95/842 (11.3) |
| Urinary disease (N39.0) | 51/842 (6.1) |
| **Psychiatric** |  |
| Depressive episode (F32.9) | 158/637 (24.8) |
| Drug abuse (F19.9) | 142/637 (22.3) |
| Anorexia (R63.0) | 73/637 (11.5) |
| **Hepatic** |  |
| Hepatic failure (K72.9) | 100/445 (22.5) |
| Ascites (R18) | 61/445 (13.7) |
| Toxic liver disease (K71.7) | 53/445 (11.9) |
| **Viral** |  |
| Acute bronchitis (J20.9) | 71/356 (19.9) |
| Gastroenteritis (A09) | 39/356 (11.0) |
| Zoster infection (B02.9) | 33/356 (9.3) |
| **Neurological** |  |
| Headache (R51) | 76/437 (17.4) |
| Dizziness (R42) | 44/437 (10.1) |
| Epilepsy (G40.9) | 43/437 (9.8) |
| **Cardiovascular** |  |
| Cerebellar stroke (G46.4) | 19/247 (7.7) |
| Cerebral infarction (I63.9) | 17/247 (6.9) |
| Hypertension (I10) | 15/247 (6.1) |
| **Parasitic** |  |
| Candidal stomatitis (B37.0) | 179/252 (71.0) |
| Mycosis (B49) | 23/252 (9.1) |
| Candidiasis of other sites (B37.8) | 9/252 (3.6) |
| **Non-AIDS malignancy** |  |
| Malignant neoplasm of the lung (C34.9) | 38/123 (30.9) |
| Malignant neoplasm, unspecified (C80) | 23/123 (18.7) |
| Hodgkin lymphoma (C81.9) | 15/123 (12.2) |

**Table S6:** Three most frequent diagnoses within the AIDS and specific non-AIDS severe morbid events categories in the IPEC Cohort.

| **Diagnosis (ICD-10 code)** | **N/N (%)** |
| --- | --- |
| **AIDS** |  |
| Toxoplasmosis (B58.2) | 161/1069 (15.1) |
| Pneumocystosis (B59) | 155/1069 (14.5) |
| Tuberculosis (A15.3) | 128/1069 (12.0) |
| **Non-AIDS** |  |
| **Bacterial** |  |
| Bacterial pneumonia (J15.9) | 283/760 (37.2) |
| Sepsis (A41.9) | 166/760 (21.8) |
| Cellulitis (L03.9) | 84/760 (11.1) |
| **Psychiatric** |  |
| Depressive episode (F32.9) | 32/90 (35.6) |
| Depressive episode with psychotic symptoms (F32.3) | 24/90 (26.7) |
| Depressive episode without psychotic symptoms (R32.2) | 14/90 (15.6) |
| **Hepatic** |  |
| Toxic liver disease with acute hepatitis (K71.2) | 85/127 (66.9) |
| Cirrhosis (K74.6) | 26/127 (20.5) |
| Chronic hepatitis C (B18.2) | 7/127 (5.5) |
| **Viral** |  |
| Gastroenteritis (A09) | 32/125 (25.6) |
| Zoster infection (B02.9) | 19/125 (15.2) |
| Non-infective gastroenteritis and colitis (K52.9) | 14/125 (11.2) |
| **Neurological** |  |
| Polyneuropathy (G62.9) | 9/13 (69.2) |
| Epilepsy (G40.5) | 2/13 (15.4) |
| Inflammatory polyneuropathy (G61.9) | 2/13 (15.4) |
| **Cardiovascular** |  |
| Congestive heart failure (I50.0) | 26/128 (20.3) |
| Thrombosis (I82.9) | 25/128 (19.5) |
| Hypertension (I10) | 24/128 (18.8) |
| **Parasitic** |  |
| Sporotrichosis (B42.7) | 9/46 (19.6) |
| Strongyloidiasis (B78.0) | 6/46 (13.0) |
| Leishmaniasis (B55.1) | 5/46 (10.9) |
| **Non-AIDS malignancy** |  |
| Malignant neoplasm of the lung (C34.9) | 2/11 (18.2) |
| Malignant neoplasm of the stomach (C16.9) | 2/11 (18.2) |
| Hodgkin lymphoma (C81.9) | 1/11 (9.1) |
